# The effects on joint functions of biplanar distal tubercle open-wedge high tibial osteotomy: A prospective study

**DOI:** 10.1097/MD.0000000000034980

**Published:** 2023-09-08

**Authors:** Sinan Zehir, Taner Alic

**Affiliations:** a Orthopaedics and Traumatology Department, Hitit University Faculty of Medicine, Corum, Turkey.

**Keywords:** biplanar, BMI, clinical outcomes, high tibial osteotomy, open-wedge, patellar height, retro-tubercle

## Abstract

Distal tubercle biplanar open-wedge high tibial osteotomy (DT-BOWHTO) is a method frequently applied in the treatment of knee joint medial osteoarthritis. The aim of this study was to evaluate the radiological, clinical, and functional results of patients at 5 years after DT-BOWHTO surgery. The study included a total of 41 patients who underwent DT-BOWHTO, comprising 19 (46.3%) males and 22 (53.7%) females with a mean age of 55.54 ± 4.17 (45–63) years and mean follow-up of 66.76 ± 6.29 (60–81) months. Statistical comparisons were made of the preoperative and postoperative body mass index (BMI), modified Insall-Salvati index, Blackburn-Peel index, Kelgren-Lawrence classification (KLC), tibial slope angle, American Knee Society Functional Score (AKSFS), Clinical American Knee Society Score (CAKSS), visual analog scale (VAS) pain score, Tegner Functional Activity Score (TFAS), total corrected angular measurements (TCA), and the tibio-femoral varus angle. Compared to the preoperative values, no statistically significant difference was determined in the postoperative modified Insall-Salvati index, Blackburn-Peel index, and tibial slope angle values (*P* > .05), and a statistically significant difference was determined in the BMI, AKSFS, CAKSS, VAS, KLC, tibio-femoral varus angle, and TFAS values (*P ≤* .001). When the preoperative and postoperative BMI values were compared in 3 groups of normal, overweight, and obese, there was found to be a statistically significant difference (*P* = .014). No significant correlation was determined between the BMI values and the VAS and KLC values (*P* > .05). No significant correlation was determined between the total corrected angular and the preoperative and postoperative pain, and clinical and functional knee scores (VAS, AKSFS, CAKSS, TFAS) (*P >* .05). DT-BOWHTO was seen to provide extremely good 5-year results in the knee clinical findings, pain severity, and functional results. As the patella height and tibial slope angle were not changed, this did not cause the development of osteoarthritis in the patellofemoral and tibiofemoral joints. Grafting and fixation of the tibial tubercle with additional screws in the application of DT-BOWHTO were not seen to make any additional contribution to the healing of the osteotomy line. There was no relationship between increased BMI, reduced pain, and increase in knee functions in patients who underwent DT-BOWHTO.

## 1. Introduction

Osteoarthritis in the medial compartment of the knee joint and associated varus malalignment is extremely commonly seen.^[1,2]^ High tibial osteotomy (HTO) is a treatment option that protects the knee joint and corrects the mechanical axis and load balance.^[1]^ By slowing down the progression of osteoarthritis with HTO, the need for total knee arthroplasty (TKA) is postponed,^[2,3]^ and by reducing the pain formed in the joint, long-term increases are provided in joint functions.^[1,2]^ Early weight-bearing after HTO allows patients to return to daily activities and sports at a high rate.^[4,5]^

HTO methods applied from the proximal tibial region are defined as 2 types; open-wedge HTO (OWHTO) and closed-wedge HTO.^[1,2,6]^ OWHTO has become more widely used in recent years as it provides more clear angular correction in both the sagittal and coronal planes, creates less peroneal nerve damage, and better clinical and functional results are obtained.^[7]^ However, it may cause patellofemoral (PF) arthritis as the patella height and the PF joint biomechanics are altered.^[1,8]^ Therefore, OWHTO modifications developed in recent years and recommended by many authors have started to be used more to prevent this. Distal tubercle biplanar open-wedge high tibial osteotomy (DT-BOWHTO), which minimizes the negative effect on the PF joint, is the leading one of these modifications and the results continue to attract interest.^[9,10]^

The aim of this prospective study was to evaluate the radiological, clinical, and functional results of patients who underwent DT-BOWHTO for medial osteoarthritis of the knee joint.

## 2. Methods

### 2.1. Patients

The study included 41 patients who underwent DT-BOWHTO with fixation with an anatomic proximal tibial locking L-plate. The patients included in the study were those with unilateral knee medial region pain that increased with movement, varus deformity of >5° on standing long-leg radiographs according to the International Cartilage Repair Society (ICRS), close to normal range of motion (flexion > 90° and flexion contracture < 5°), had at least 5 years of follow up, no history of arthroscopic surgery on the knee, no anterior cruciate ligament rupture, no fracture or additional injury around the knee, and a femoral medial chondral lesion ≤ 3° applied with microfracture in the first arthroscopy. The study exclusion criteria were defined as the presence of infection around the knee, bicompartmental osteoarthritis, rheumatoid arthritis, periarticular fracture, severe varus deformity (>15°), knee range of motion < 90°, vascular or neurological disorder, a history of HTO, or refusal to provide informed consent.

All the patients included in the study underwent a detailed clinical examination, standing posteroanterior and lateral radiographs, and magnetic resonance imaging to discount bicompartmental gonarthrosis. The degree of varus deformity was determined from mechanical and anatomic axis measurements taken on the direct radiographs. The preoperative, clinical follow-up, and final evaluation data of the patients were recorded. The data examined were the values of body mass index (BMI), modified Insall-Salvati index (MISI), Blackburn-Peel index (BPI), Kelgren-Lawrence classification (KLC), tibial slope angle (TSA), American Knee Society Functional Score (AKSFS), Clinical American Knee Society Score (CAKSS), visual analog scale (VAS) pain score, Tegner Functional Activity Score (TFAS), total corrected angular (TCA) measurements and the tibio-femoral varus angle (TFVA). Complications that could develop were followed up and recorded and the cases that were converted to TKA. Statistical comparisons were made of the preoperative and postoperative numerical data of the radiological angular measurements, rates and scores.

### 2.2. Surgical technique

With the patient positioned supine on the operating table, anesthesia was administered then sterile conditions were provided for a graft to be taken from the ipsilateral iliac wing. Following application of a tourniquet, the joint was evaluated arthroscopically. The necessary debridement was performed for problems such as osteochondritis dissecans, degenerative torn meniscus, or synovial hypertrophy. For a femoral medial condyle chondral lesion, microfracture was performed at 1 mm diameter and 10 mm depth. Patients with an advanced stage cartilage lesion were excluded from the study as osteotomy could not be performed.

**Figure 1.**
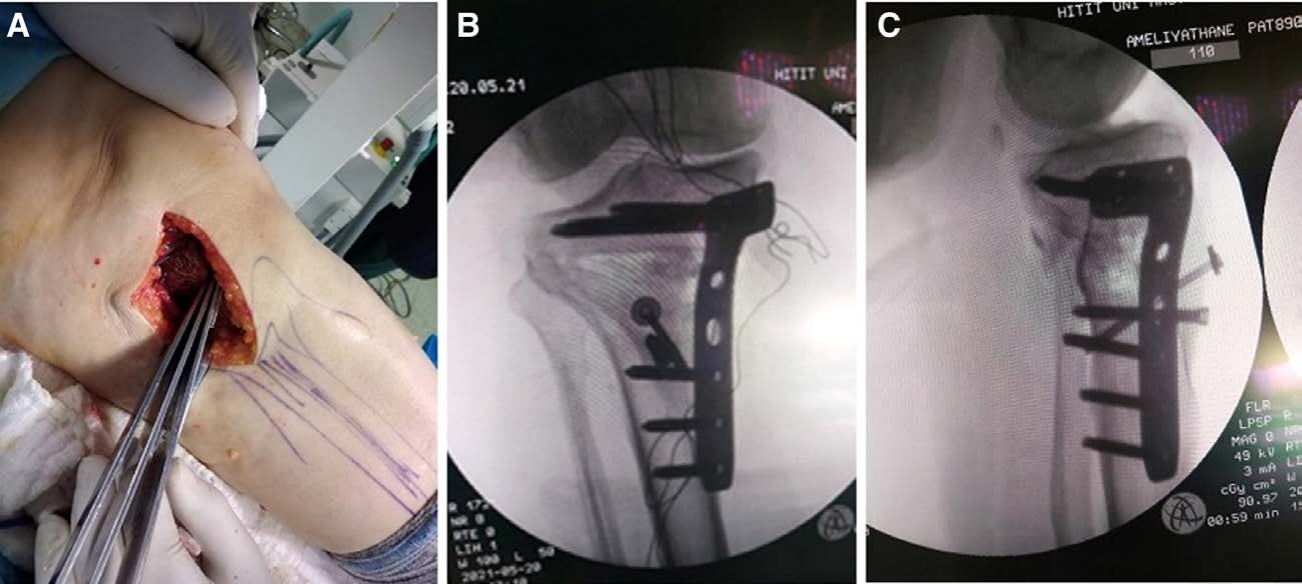
Adjusting the total correction angle with tibial osteotomy. (A) After fixation of the tibial tubercle with 2 additional screws, (B) anterior–posterior fluoroscopy images, (c) lateral fluoroscopy images.

The fascia was opened with an approximately 7 to 9 cm longitudinal incision made immediately anterior to the medial collateral ligament in the anteromedial of the proximal tibia. The surface part of the medial collateral ligament was stripped from the bone towards the posterior. Under fluoroscopy guidance, 2 Kirschner wires (K-wires) were advanced parallel to the joint towards the lateral cortex at an appropriate angle to target the proximal fibula. That the osteotomy line would be 3 to 4 cm distal of the joint line was checked under fluoroscopy. A biplanar retrotubercle osteotomy was performed in accordance with the described technique in horizontal and close to vertical oblique directions with the first 2 to 3 cm of the cut made with a motorized saw from the medial cortex towards the lateral under K-wire guidance.^[10,11]^ Then by expanding with osteotomes, anterior and posterior cortex cuts were made without breaking the lateral cortex. The osteotomy line was distracted by applying an expander in the form of a wedge to the osteotomy line without removing the K-wires. The lower extremity mechanical axis was evaluated with the cable test and the appropriate angular correction was provided. After removal of the distractor, the K-wires prevented position loss and breakage in the lateral cortex. Fixation was applied with an L-type 4.5 mm proximal tibia plate (Tibial proximal L-plates, Ortomega Medical Engineering Ltd., Izmir, Turkey). Proximal and distal fixation was provided with screws over the plate, and additional fixation to the tibial tubercle was made with 2 screws (Fig. 1). The fixations were checked under fluoroscopy, then grafting was applied to the osteotomy line with the autograft taken from the iliac wing. A hemovac drain for free drainage was applied to each patient.

In the postoperative early period, joint movement with a continuous passive motion device (Rimec Fisiotek 3000 E for knee and hip CPM- Erke Medical) and active quadriceps strengthening exercises were started. For mobilization during the first 6 weeks, a hinged brace on the knee and crutches were used. Then partial weight-bearing as tolerated was permitted with gradual increases until full weight-bearing after bone union.

No intraoperative complications developed in any patient, such as tubercle fracture, vascular nerve damage, or lateral cortex fracture. There were also no early postoperative complications such as wound site problems, fixation failure, nonunion, deep vein thrombosis, pulmonary thromboemboli, or conversion to TKA.

### 2.3. Radiographic method

All the radiological measurements were performed by an orthopedist blinded to the study. The functional score and measurements were formed of the values recorded preoperatively and at the final follow-up evaluation. The radiological evaluations were made on the radiographs taken preoperatively and at the final follow-up examination. These were standing long-leg anterior–posterior radiographs, and lateral views in 30° flexion and neutral position.

Coronal alignment was evaluated with the medial proximal tibial angle and the mechanical femorotibial angle (values showing valgus and varus) as described by Paley and Tetsworth.^[12]^ The femoral mechanical axis passes from the hip center to the knee center, and the tibial mechanical axis passes from the center of the tibial spine to the center of the ankle. The angle formed between these 2 axes is the mechanical femorotibial angle. Patellar height was evaluated on the standing knee lateral image with the measurements of the Insall-Salvati Index,^[13]^ MISI,^[14]^ and BPI.^[15]^ The tibial slope was calculated with the proximal tibial anatomic axis method by measuring the angle formed between the line drawn vertical to the tibial shaft axis and the proximal medial plateau.

### 2.4. Statistical analysis

Data obtained in the study were analyzed statistically using SPSS vn. 22.0 software (SPSS Inc., Chicago, IL). Conformity of the data to normal distribution was assessed with the Shapiro–Wilk test. In the reporting of descriptive statistics, continuous variables were stated as mean ± standard deviation (SD) or median (minimum–maximum) values, and categorical variables as number (n) and percentage (%). In the comparisons of the preoperative and postoperative measurements, the Paired Samples *t*-test was applied to dependent groups of variables showing normal distribution, and the Wilcoxon Signed Rank test to data not showing normal distribution. The McNemar-Bowker test was used in the comparisons of rates and relationships between categorical variables. Correlations between numerical variables were analyzed using the Spearman correlation coefficient. A value of *P* < .05. was accepted as statistically significant.

## 3. Results

Evaluation was made of a total of 41 patients who underwent DTO, comprising 19 (46.3%) males and 22 (53.7%) females with a mean age of 55.54 ± 4.17 (range, 45–63) years and mean BMI of 28.54 ± 4.37 kg/m^2^. The mean follow-up period was 66.76 ± 6.29 (range, 60–81) months (Table 1).

**Table 1:** Descriptive statistics on demographic and clinical characteristics of patients (N = 41).

		Data
Gender	Male	19 (46.3)
	Female	22 (53.7)
Side	Right	13 (31.7)
	Left	28 (68.3)
Age		55.54 ± 4.17 (45–63)
Union time (week)		13.98 ± 1.47 (11–17)
Follow-up time (month)		66.76 ± 6.29 (60–81)
TCA (°)		13.95 ± 1.44 (10–17)
Operation time (minute)		64.49 ± 7.12 (45–80)

Data are presented as mean ± standard deviation and n (%).

SD = standard deviation, TCA = total correction angle.

**Figure 2.**
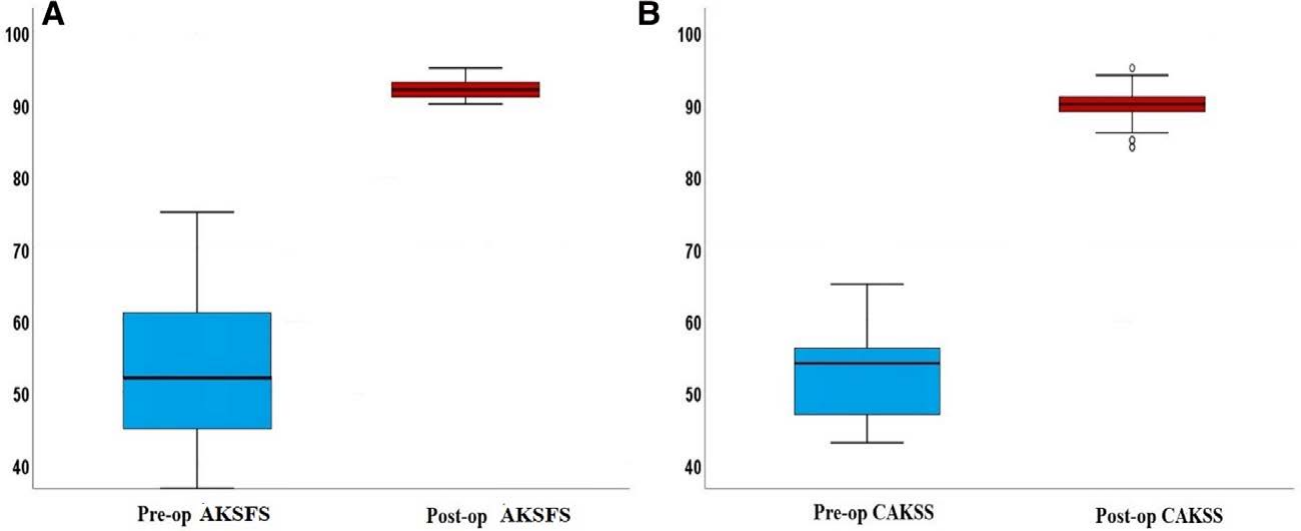
Box plot showing preoperative and postoperative comparison of Functional Knee Score and Clinical American Knee Society Score knee scores and angles.

**Figure 3.**
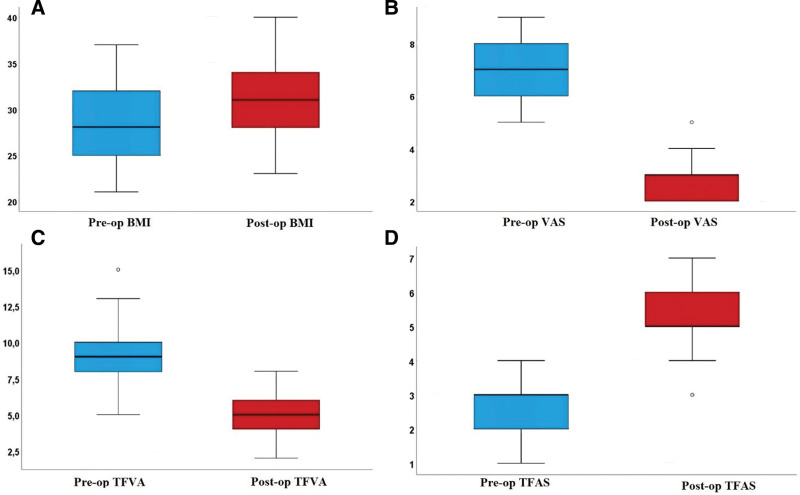
Box plot showing preoperative and postoperative comparison of body mass index (A), visual analog scale (B), tibio-femoral varus angle (C), Tegner Functional Activity Score (D). Image of the determination of the tibial proximal anatomical axis.

The results of the comparisons of the preoperative and postoperative values of MISI, BPI, TSA, BMI, AKSFS, CAKSS, VAS, KLC, TFVA, and TFAS are shown in Table 2. Compared to the preoperative values, no statistically significant difference was determined in the postoperative MISI, BPI, and TSA values (*P* = .317, *P* = 1.000, *P* = .763, respectively) (Table 2). The differences between the preoperative and postoperative AKSFS, CAKSS (Fig. 2), BMI, VAS, TFVA, TFAS (Fig. 3), and KLC values were determined to be statistically significant (*P =* .001 for all) (Table 2).

**Table 2:** Preoperative and postoperative comparison of knee scores and angles.

	Preoperative	Postoperative	*P*
MISI	1 (1–2)	1 (1–2)	.317*
BPI	1 (1–1)	1 (1–1)	1.000*
TSA (°)	8 (3–11)	8 (4–11)	.763*
BMI (kg/m^2^)	28 (21–37)	31 (23–40)	.001*
AKSFS	52.68 ± 11.47	92.07 ± 1.47	<.001†
CAKSS	52.54 ± 5.69	89.80 ± 2.61	<.001†
VAS	7 (5–9)	3 (2–5)	<.001*
KLC	3 (2–4)	2 (1–3)	<.001*
TFVA (°)	8.83 ± 2.25	-5.12 ± 1.47	<.001†
TFAS	3 (1–4)	5 (3–7)	<.001*

Data are presented as mean ± standard deviation and median (min–max)

AKSFS = American Knee Society Functional Score, BMI = body mass index, BPI = Blacburn-Peel index, CAKSS = Clinical American Knee Society Score, KLC = Kelgren-Lawrence classification, MISI = modifiye Insal Salvati index, TFAS = Tegner Functional Activity Score, TFVA = tibio-femoral varus angle, TSA = tibial slope angle, VAS = visual analog scale.

* Paired *t* test with.

† Wilcoxon signed rank.

When the preoperative and postoperative BMI values were compared in 3 groups of normal, overweight, and obese, there was found to be a statistically significant difference (*P* = .014) (Table 3).

**Table 3:** Ratio comparison between preoperative and postoperative body mass index.

		Postoperative BMI (kg/m^2^)			Total	*P*
		<24.9	25–29.9	>30		
Preoperative BMI (kg/m^2^)	<24.9	1 (2.4)	4 (9.8)	5 (12.2)	10 (24.4)	.014
	25–29.9	0	7 (17.1)	7 (17.1)	14 (34.1)	
	>30	0	3 (7.3)	14 (34.1)	17 (41.5)	
Total		1 (2.4)	14 (34.1)	26 (63.4)	41 (100)	

Data are presented as n (%).

McNemar-Bowker test.

BMI = body mass index.

No significant correlation was determined between the preoperative and postoperative BMI values and the VAS and KLC values (*P* > .05) (Table 4).

**Table 4:** Correlation analysis findings between preoperative and postoperative body mass index values and preoperative, postoperative visual analog scale, Kelgren-Lawrence classification, American Knee Society Functional Score, Clinical American Knee Society Score, Tegner Functional Activity Score values.

			BMI	
			Preoperative	Postoperative
VAS	Preoperative	r	.108	–.132
		*P*	.503*	.410*
	Postoperative	r	–.139	–.135
		*P*	.385*	.402*
KLC	Preoperative	r	–.180	–.219
		*P*	.261	.168*
	Postoperative	r	.137	.123
		*P*	.393*	.444*
AKSFS	Preoperative	r	.397	.182
		*P*	.010*	.254*
	Postoperative	r	–.189	–.122
		*P*	.238*	.449*
CAKSS	Preoperative	r	–.212	–.125
		*P*	.183*	.435*
	Postoperative	r	–.259	.023
		*P*	.102*	.887*
TFAS	Preoperative	r	.099	–.065
		*P*	.536†	.689†
	Postoperative	r	.068	–.070
		*P*	.674†	.663†

AKSFS = American Knee Society Functional Score, BMI = body mass index, CAKSS = Clinical American Knee Society Score, KLC = Kelgren-Lawrence classification, TFAS = Tegner Functional Activity Score, VAS = visual analog scale.

* Person correlation analysis.

† Spearman correlation coefficient.

The correlation analysis findings between preoperative and postoperative BMI values and preoperative and postoperative TFAS, AKSFS, and CAKSS values are presented in Table 4. No statistically significant correlation was found between preoperative BMI and TFAS, CAKSS values, and between postoperative BMI values and TFAS, AKSFS, CAKSS values (*P* > .05). There was a positive and weak statistically significant correlation between preoperative BMI and preoperative AKSFS values (r = .397; *P =* .010).

No significant correlation was determined between the TCA and the preoperative and postoperative knee functional scores (*P* > .05) (Table 5).

**Table 5:** Findings of correlation analysis between total correction angle and preoperative and postoperative values of functional scores.

			TCA
AKSFS	Preoperative	r	–.209
		*P*	.190
	Postoperative	r	–.061
		*P*	.704
CAKSS	Preoperative	r	–.072
		*P*	.656
	Postoperative	r	–.145
		*P*	.365
VAS	Preoperative	r	–.245
		*P*	.123
	Postoperative	r	.031
		*P*	.850
TFAS	Preoperative	r	–.247
		*P*	.119
	Postoperative	r	–.173
		*P*	.279

Pearson correlation analysis.

AKSFS = American Knee Society Functional Score, CAKSS = Clinical American Knee Society Score, TCA = total correction angle, TFAS = Tegner Functional Activity Score, VAS = visual analog scale.

## 4. Discussion

The most significant findings of this study were that significant positive effects were seen in the knee pain and function parameters (AKSFS, CAKSS, VAS, KLC, TFVA, TFAS) of patients following DT-BOWHTO surgery for knee joint medial osteoarthritis. No negative effect was determined in patellar height, PF joint, tibial slope, or in the tibiofemoral joint (MISI, BPI, TSA). Although there was a significant rate of weight increase in the patients, this increase was not seen to be associated with a decrease in pain, regression in osteoarthritis, or recovery of knee functions. The knee function scores and clinical improvement scores were not found to be associated with the degree of the total correction angle.

Changes in BMI after DT-BOWHTO surgery and the effect of this on the surgical results is a subject of interest. To the best of our knowledge, there is no prospective mid or long-term study in the literature showing BMI changes. Therefore, there can be considered to be a need for such a study. The mean BMI of the patients included in the current study was 28.5 kg/m^2^ preoperatively, and 31.1 kg/m^2^ at the final follow-up examination. Of the 10 patients with a preoperative BMI of 18 to 25 kg/m^2^, only 1 patient remained at this value at the final follow-up, whereas 6 patients had progressed to BMI 25 to 30 kg/m^2^, and 3 patients to BMI > 30 kg/m^2^. The 14 patients with preoperative BMI 25 to 30 kg/m^2^ remained at this level, with the addition of the 6 who progressed to this value. The number of patients with BMI > 30 kg/m^2^ increased from 17 to 26. Thus in the postoperative period there was a significant increase in weight in the patients, but this increase in BMI was not determined to be related to a decrease in pain, or increase in clinical status and functions. In other words, the clinical and functional improvements were independent of BMI values.

Herbst M. et al^[16]^ evaluated BMI, conversion to TKA, and complications that developed in patients who underwent OWHTO, and unlike the current study obtained lower mid-term clinical and functional results in overweight patients with high BMI. It was also determined that independently of BMI values, the rate of conversion to TKA was approximately 10.5%. There was not determined to be any significant relationship between BMI and the development of major complications (arthrofibrosis, thrombosis, pulmonary emboli, implant failure, surgical site infection, hematoma requiring additional surgery).^[16]^ In the current study, there were no complications and no conversions to TKA in any patient. Therefore, no correlation was determined between BMI and the development of complications or conversion to TKA. Similarly, in a study with a 2-year follow-up, Wu et al^[17]^ reported that there was no correlation between BMI values and conversion to TKA, the development of complications, or clinical, functional, and radiological healing.

Another subject of debate following HTO operations is the effect on the PF joint of the change in patellar tendon length and patella height. This problem is seen more often especially after HTO operations performed in a single plane.^[1,8,18]^ However, DT-BOWHTO is a technique which provides patellar tendon length stability, prevents PF joint problems, and has good results reported.^[18,19]^ Different indexes can be used in the evaluation of PF height. As the MISI and BPI are accepted as indexes that are effective in showing patella height,^[1]^ these 2 indexes were used in the current study. TCA in the HTO operation and preoperative patella height are important markers of PF osteoarthritis.^[1]^ In a study in which changes in pressure in the PF joint were evaluated, Kloos et al^[10]^ showed that angular corrections beyond 15° with monoplanar HTO increased contact pressure in the PF joint, but caused lower contact pressures after HTO and DT-BOWHTO. In the current study, although the mean TCA was 14° (10–17°), no findings of PF osteoarthritis were determined. It can be considered that in larger patient series there may be a TCA cutoff value in the development of PF osteoarthritis, and this warrants investigation. Stofell et al^[20]^ reported that the normal joint biomechanics were protected in medial open-wedge high tibial osteotomy and biplanar DT-BOWHTO, and there was concluded to be no significant effect on PF contact pressures. Lee et al^[21]^ stated that overcorrection after medial open-wedge high tibial osteotomy could induce PF degeneration and could lead to poor results in respect of the PF joint. In another study, Sim et al^[22]^ reported a significant decrease in patella height and significant arthritic changes in the PF joint at 2 years after OWHTO. At mean 21 to 32 months after HTO, Kim et al^[23]^ reported PF osteoarthritis at the rate of 21% to 41% in second arthroscopy performed at the same time as plate removal. However, patella height was not affected following biplanar distal tubercle osteotomy.^[1]^ Consistent with the findings in literature, PF osteoarthritis did not develop in any of the current study patients following DT-BOWHTO. As there was no change in patella height from preoperative to postoperative, there was no difference in the MISI and BPI values. Moreover, as the patients were followed up for mean 67 (range, 60–81) months in this study, the long-term effects after DT-BOWHTO were shown. Nevertheless, there is a need for further studies with more biomechanical tests to clarify the biomechanical basis of this.

Changes in the TSA affect the translation forces in the knee and alter anterior–posterior knee stability.^[22]^ Previous biomechanical studies have shown that an increase in TSA decreases the loading on the posterior cruciate ligament, and a decrease in TSA decreases the loading on the anterior cruciate ligament.^[23]^ Kyung et al^[24]^ reported that changes of up to 5° in TSA values were not clinically significant. In the current study, there was no significant change in the TSA values from preoperative to postoperative (7.78–7.8°). Thus both the anterior and posterior cruciate ligaments were protected and no change was determined in the anterior–posterior stability of the knee. The TFVA value in the current study was mean 8.88° in varus preoperatively and –5.12° in valgus after the osteotomy. Although the TCA was 13.9°, no correlation was determined with the clinical, functional, or pain scores. In 2 different studies where the mean TCA was 11.5°, good clinical and radiological results were reported.^[2,24]^ Although no cutoff value of the TCA value could be determined to show the effect on knee functions in the current study, it could be possible with greater numbers of patients in future studies. Consistent with the literature, a significant clinical and functional increase was determined after DT-BOWHTO, and a decrease in osteoarthritis and pain, and therefore, no patient required conversion to TKA.

Complications during the HTO operation,^[25]^ tendinitis which can develop later,^[26]^ and the management of healing problems are thought to be important. Türkmen et al^[27]^ reported that the biplanar method was better than the monoplanar method in respect of lateral cortex fracture in the proximal tibia. Despite using no additional fixation to the tibial tubercle in that study, no complications were encountered such as tibial plateau fracture or tibial tubercle fracture.^[26]^

Although single screw fixation of the tibial tubercle after biplanar osteotomy can cause additional problems such as tubercle fracture, it is effective in the union of the osteotomy region,^[6]^ and is recommended for additional stabilization to reduce the risk of tubercle fracture and delayed union.^[2]^ In the current study, in addition to plate-screw fixation, 2 more screws were applied to the tibia tubercle, and this was thought to have contributed to stability both in the osteotomy line and in the tibial tubercle. Although the time to union was similar to values reported in literature (mean 14 weeks), fixation in 2 different planes in the biplanar osteotomy can provide rotational stability. However, to support this view, there is a need for biomechanical studies.

Grafting is another debatable point in the union of the osteotomy line. Some researchers have claimed that grafting is not necessary in open wedge distance of <10 mm, and others have reported that there can be effective union without any fixation applied.^[2]^ In the current series, grafting from the iliac wing was performed in all cases, and weight-bearing was determined in the 6th week. However, no positive effect of grafting was determined on union. As one patient developed superficial infection in the graft donor site in the iliac wing, it was concluded that grafting may not be necessary because of potential complications.

The power of this study was slightly reduced by limitations such as the absence of a control group, that although long-term results were presented the sample size was relatively small, and that the results were not supported with biomechanical measurements. A further limitation could be said to be that no cutoff values could be determined of the angular measurements causing change in the functional scores.

## 5. Conclusion

DT-BOWHTO is a method applied in the treatment of knee medial osteoarthritis, which has low complication rates and prevents the need for arthroplasty. The 5-year clinical and functional results and pain severity in the knee joint were seen to be extremely good. As the patella height and tibial slope angles do not change after DT-BOWHTO, it does not cause the development of osteoarthritis in the PF and tibiofemoral joints. Grafting and fixation of the tibial tubercle with additional screws in the application of DT-BOWHTO do not provide any additional contribution to the healing time of the osteotomy line. The increase in BMI determined in the patients who had undergone DT-BOWHTO was not found to be related to decreased pain and increased knee functions.

## Acknowledgments

The authors thank Emre Demir for the medical statistics.

## Author contributions

**Conceptualization:** Sinan Zehir, Taner Alic.

**Data curation:** Sinan Zehir, Taner Alic.

**Formal analysis:** Sinan Zehir, Taner Alic.

**Funding acquisition:** Sinan Zehir, Taner Alic.

**Investigation:** Sinan Zehir, Taner Alic.

**Methodology:** Sinan Zehir, Taner Alic.

**Project administration:** Sinan Zehir, Taner Alic.

**Resources:** Sinan Zehir, Taner Alic.

**Software:** Sinan Zehir, Taner Alic.

**Supervision:** Sinan Zehir, Taner Alic.

**Validation:** Sinan Zehir, Taner Alic.

**Visualization:** Sinan Zehir, Taner Alic.

**Writing – original draft:** Sinan Zehir, Taner Alic.

**Writing – review & editing:** Taner Alic.

## References

[1] Kurien T East J Mandalia V. The effects of open wedge high tibial osteotomy for knee osteoarthritis on the patellofemoral joint. A systematic review. Knee. 2023;40:201–19. 36512892 10.1016/j.knee.2022.11.023

[2] Kornah B Abdel-Hameed SK Abdel-AAl M. Biplanar open-wedge high tibial osteotomy with locking plate for treatment of osteoarthritic varus knee. Open J Orthop. 2019;09:1–13.

[3] Ollivier B Berger P Depuydt C. Good long-term survival and patient-reported outcomes after high tibial osteotomy for medial compartment osteoarthritis. Knee Surg Sports Traumatol Arthrosc. 2021;29:3569–84. 32909057 10.1007/s00167-020-06262-4

[4] Schröter S Ateschrang A Löwe W. Early full weight-bearing versus 6-week partial weight-bearing after open wedge high tibial osteotomy leads to earlier improvement of the clinical results: a prospective, randomised evaluation. Knee Surg Sports Traumatol Arthrosc. 2017;25:325–32. 25854499 10.1007/s00167-015-3592-x

[5] Ekhtiari S Haldane CE de Sa D. Return to work and sport following high tibial osteotomy: a systematic review. J Bone Joint Surg Am. 2016;98:1568–77. 27655985 10.2106/JBJS.16.00036

[6] Park SB Kim JS Jeong HW. Medially and distally inserted tuberosity screw fixation of the osteotomized tubercle is safe and effective in retro-tubercular bi-planar opening-wedge high tibial osteotomy. Knee Surg Sports Traumatol Arthrosc. 2023;31:1571–82. 35680679 10.1007/s00167-022-07009-z

[7] Gaasbeek RD Nicolaas L Rijnberg WJ. Correction accuracy and collateral laxity in open versus closed wedge high tibial osteotomy. A one-year randomised controlled study. Int Orthop. 2010;34:201–7. 19707760 10.1007/s00264-009-0861-7 PMC2899362

[8] Kloos F Becher C Fleischer B. High tibial osteotomy increases patellofemoral pressure if adverted proximal, while open-wedge HTO with distal biplanar osteotomy discharges the patellofemoral joint: different open-wedge high tibial osteotomies compared to an extra-articular unloading device. Knee Surg Sports Traumatol Arthrosc. 2019;27:2334–44. 30291397 10.1007/s00167-018-5194-x

[9] Kim SJ Mahajan RH Park KY. Biplanar medial open-wedge high tibial osteotomy for medial compartment osteoarthritis of the knee: a novel technique and follow-up. Oper Tech Orthop. 2007;17:29–37.

[10] Esenkaya I Unay K. Proximal medial tibial biplanar retrotubercle open wedge osteotomy in medial knee arthrosis. Knee. 2012;19:416–21. 21561777 10.1016/j.knee.2011.03.009

[11] Esenkaya I Elmali N Misirlioğlu M. Proksimal tibia medial açik kama osteotomisinde lateral plato kiriği oluşumunu önlemek için alternatif uygulama. Dana tibialarinda deneysel çalişma. İnönü Üniversitesi Tip Fakültesi Derg. 2005;12:71–5.

[12] Paley D Tetsworth K. Mechanical axis deviation of the lower limbs. Preoperative planning of uniapical angular deformities of the tibia or femur. Clin Orthop Relat Res. 1992:48–64. 1611764

[13] Insall J Salvati E. Patella position in the normal knee joint. Radiology. 1971;101:101–4. 5111961 10.1148/101.1.101

[14] Grelsamer RP Meadows S. The modified Insall-Salvati ratio for assessment of patellar height. Clin Orthop Relat Res. 1992:170–6. 1516309

[15] Blackburne JS Peel TE. A new method of measuring patellar height. J Bone Joint Surg Br. 1977;59:241–2. 873986 10.1302/0301-620X.59B2.873986

[16] Herbst M Ahrend MD Grünwald L. Overweight patients benefit from high tibial osteotomy to the same extent as patients with normal weights but show inferior mid-term results. Knee Surg Sports Traumatol Arthrosc. 2022;30:907–17. 33570698 10.1007/s00167-021-06457-3 PMC8901480

[17] Wu CY Huang JW Lin CH. Preoperative overweight and obesity do not cause inferior outcomes following open-wedge high tibial osteotomy: a retrospective cohort study of 123 patients. PLoS One. 2023;18:e0280687. 36662878 10.1371/journal.pone.0280687 PMC9858777

[18] Turkmen I Esenkaya I. A patellar tendon length conservation method: biplanar retrotubercle open-wedge proximal tibial osteotomy. North Clin Istanb. 2018;5:246–53. 30688937 10.14744/nci.2018.52243 PMC6323570

[19] Elmali N Esenkaya I Can M. Monoplanar versus biplanar medial open-wedge proximal tibial osteotomy for varus gonarthrosis: a comparison of clinical and radiological outcomes. Knee Surg Sports Traumatol Arthrosc. 2013;21:2689–95. 22644071 10.1007/s00167-012-2040-4

[20] Stoffel K Willers C Korshid O. Patellofemoral contact pressure following high tibial osteotomy: a cadaveric study. Knee Surg Sports Traumatol Arthrosc. 2007;15:1094–100. 17342550 10.1007/s00167-007-0297-9

[21] Lee SS So SY Jung EY. Predictive factors for patellofemoral degenerative progression after opening-wedge high tibial osteotomy. Arthroscopy. 2019;35:1703–10. 31053459 10.1016/j.arthro.2019.01.032

[22] Sim JA Na YG Lee BK. Alignment changes after open-wedge high tibial osteotomy result in offloading in the patellofemoral joint: a SPECT/CT analysis. Knee Surg Sports Traumatol Arthrosc. 2022;30:437–46. 32577783 10.1007/s00167-020-06115-0

[23] Kim KI Kim DK Song SJ. Medial open-wedge high tibial osteotomy may adversely affect the patellofemoral joint. Arthroscopy. 2017;33:811–6. 28043753 10.1016/j.arthro.2016.09.034

[24] Kyung HS Lee BJ Kim JW. Biplanar open wedge high tibial osteotomy in the medial compartment osteoarthritis of the knee joint: comparison between the Aescula and TomoFix plate. Clin Orthop Surg. 2015;7:185–90. 26217464 10.4055/cios.2015.7.2.185 PMC4515458

[25] Turgut A Kayali C Ağuş H. Radiological analysis of closed-wedge high tibial osteotomy. Eklem Hastalik Cerrahisi. 2012;23:82–7. 22765486

[26] Bedrettin A Osman YM. Incidence of adductor tubercle tendinitis and its effect on clinical results in patients with genu varum undergoing high tibial osteotomy. Eur Rev Med Pharmacol Sci. 2023;27:144–50. 36647862 10.26355/eurrev_202301_30865

[27] Türkmen F Kaçira BK Özkaya M. Comparison of monoplanar versus biplanar medial opening-wedge high tibial osteotomy techniques for preventing lateral cortex fracture. Knee Surg Sports Traumatol Arthrosc. 2017;25:2914–20. 26897136 10.1007/s00167-016-4049-6

